# Miscellaneous Notices

**Published:** 1855-01

**Authors:** 


### MISCELLANEOUS NOTICES.

*Vermont Society of Dental Surgeons.*—As will be seen by the following report of proceedings, furnished by the Secretary, a society of dental surgeons has recently been organized in Vermont. The senior editor had the pleasure of meeting the members of the association on the 25th of November last, the day of the permanent organization of the society, and from the *esprit du corps* exhibited on the occasion, he anticipates from their labors the most beneficial results. The only feeling manifested on the occasion, so far as he could perceive, was a desire to advance the cause of science and elevate the dignity of the dental profession. That their efforts may be crowned with the fullest success is most ardently to be desired. He has received within the last few days a copy of the constitutions, by-laws, and rules of order, adopted by the association, and regrets that he has not room to publish them in the present number of the Journal. They shall appear in the next.—SEN. ED.

*Report of the Formation and Proceedings of the Vermont Society of Dental Surgeons.*—In pursuance of a call, the members of the dental profession in this state, met at the Central House in Brattleboro, September 12, 1854. On motion to that effect, O. R. Post was appointed Chairman, and E. H. Kilbourne, Secretary. The objects of the meeting were then stated to the convention by the Chairman. A general discussion then ensued in relation to the expediency of forming a permanent dental organization for the mutual improvement of its members in this state, and elevation of the profession in general.

On motion of E. H. Kilbourne, a committee was appointed to draft a constitution and by-laws for a permanent organization and report at an adjourned meeting to be held at Montpelier on Wednesday and Thursday, October 25th and 26th. The following gentlemen were appointed on said committee: N. G. Hale, Windsor; O. R. Post, Brattleboro; M. Newton, Montpelier; F. J. Hendee, Burlington; I. D. Kilbourne, St. Johnsbury; E. V. N. Harwood, Rutland; H. S. Chase, Woodstock.

On motion, M. Newton was appointed a committee to correspond with dental professors relative to procuring a lecture to be delivered before the society at its next meeting in October.

A vote was taken and carried, that the chairman of the committee issue a circular setting forth the proceedings of this meeting, and stating that a lecture may be expected at the meeting in October. An earnest desire was expressed that every member of the profession in regular standing might be present.

On motion of E. H. Kilbourne, it was voted that H. S. Chase, of Woodstock, be requested to prepare and deliver the opening address before the society in October next.

Agreeably to adjournment the society met at Montpelier, at 10 o'clock, A. M., on the 25th of October, at the office of G. H. Kilbourne. A large number of the members of the profession in the state was present.

The meeting was called to order by the President, O. R. Post.

The committee appointed at a previous meeting to draft a constitution and by-laws, reported the result of their labors. The report was accepted.

The committee to procure a lecturer reported, that Prof. Chapin A. Harris, M. D., D. D. S., of the Baltimore Dental College, had accepted an invitation to deliver a lecture before the society, and that the use of the Free Church had been secured for the occasion.

On motion, the same committee was instructed to have a printed notice circulated, inviting the public to attend the lecture at the Free Church, at 7 o'clock, P. M.

On motion, the constitution and by-laws reported by the committee were taken up, article by article, for adoption.

After some amendments, the constitution, by-laws and rules of order, were unanimously adopted.

The President said the next business was the election of members, when E. V. N. Harwood, of Rutland; O. P. Forbush, of Montpelier; J. Brockway, of Middlebury; F. M. Perry, of Barton; J. D. Kilbourne, of St. Johnsbury; Ira R. Piper, of Montpelier; G. H. Kilbourne, of Mountpelier; L. D. Thompson, of St. Albans; L. F. Gallup, of Woodstock; H. L

Williams, of Pomfret; A. M. Mowe, of Lebanon; N. H. & H. D. Smith, of Williston, were elected and became members.

Prof. C. A. Harris, M. D., D. D. S., of Baltimore, Md.; Prof. J. D. White, M. D., D. D. S., of Philadelphia, Pa.; Prof. A. Westcott, M. D., D. D. S., of Syracuse, N. Y.; and C. R. Coffin, D. D. S., of Portland, Me., were elected honorary members.

The election of officers then took place, when the following gentlemen were elected: O. R. Post, of Brattleboro, President; M. Newton, of Montpelier, 1st Vice-president; Josephus Brockway, of Midddlebury, 2d Vice-president; E. V. N. Harwood, of Rutland, Recording Secretary; N. G. Hale, Corresponding Secretary; E. H. Kilbourne, of St. Johnsbury, Treasurer, G. H. Kilbourne, of Montpelier, Librarian.

L. Gillman, J. D. Kilbourne, O. P. Forbush, Executive Committee.

G. H. Kilbourne, F. J. Hendee, M. Newton, Examining Committee.

H. S. Chase and J. Brockway, were appointed to read essays; and G. H. Kilbourne chosen to deliver the opening address at the next annual meeting.

At the appointed hour in the evening the Free Church was filled with an intelligent audience, who listened to the lecture of Dr. Harris with manifest interest. The subject of the lecture was the advantages of Association.

After the lecture a social meeting was held at the rooms of Newton and Forbush, at which Prof. Harris and Dr. Coffin were present, and made some statements on the comparative merits of crystallized gold and gold foil for filling teeth. The meeting was one of lively interest, and held to a late hour.

Thursday, 10 o'clock, A. M.—The 1st Vice-president called the meeting to order.

On motion, committees were appointed to report at the next annual meeting on the following subjects:

On the merits of crystallized gold for filling teeth; committee, M. Newton, E. V. N. Harwood, J. D. Kilbourne, H. H. Newton and O. R. Post.

On the best manner of treating exposed nerves preparatory to filling the teeth; committee, G. H. Kilbourne, F. M. Perry, O. P. Forbush, H. D. Smith and L. D. Thompson.

On diseases of the gums and their treatment; committee, M. Newton, L. F. Gallup, E. H. Kilbourne, H. H. Newton, A. D. Putnam, F. J. Hendee and L. Gillman.

On the purity and manufacture of gold foil; committee, O. P. Forbush, L. F. Gallup and J. D. Kilbourne.

The following resolutions were adopted:

***Resolved,*** That this Society tenders to Prof. Harris the expression of its thanks for his very able and interesting lecture; with a request for its publication in the American Journal of Dental Science.

*Resolved,* That the Recording Secretary be requested to prepare an abstract of the proceedings of the Society for publication in the American Journal of Dental Science, the Dental News Letter and New York Dental Recorder.

On motion, adjourned to meet at Montpelier, on the 4th Wednesday of October, 1855.

*Rutland, Nov. 20th,* 1854.

---

*Associated Alumni of Dental Colleges.*—The minutes of the proceedings of this Association should have appeared in the April number of the Journal, but were not furnished in time for that issue, and were inadvertently left out of the July number. The following is a brief outline of them.

The second annual meeting was held March 2d, 1854. It was called to order at 10 A. M., by the President, Professor A. A. Blandy. Professor Townsend delivered the opening address. Dr. Harris read a paper on the diseases of the dental pulp; Dr. W. H. Dwinelle read one on the history and application of the microscope. Dr. M. D. French read a report on mechanical dentistry. Prof. Blandy presented a paper on obturators, which was ordered to be published with the papers above noticed. A Constitution and By-Laws, after various amendments, were adopted.

The following gentlemen were elected members, viz. Drs. J. W. Derr, S. D. French, S. T. De Graffenreid, J. R. Grant, E. N. Harris, E. L. Ligon, H. W. Mason, W. Russell, R. F. Taylor, W. A. Williams, L. N. Cochran. The following gentlemen were also proposed and elected honorary members, Drs. F. H. Badger, H. Faville and C. Johnston.

The following gentlemen were elected officers for the ensuing year: Dr. W. W. H. Thackston, President; Drs. W. H. Dwinelle and C. W. Ballard, Vice-presidents; Dr. A. S. Piggot, Corresponding Secretary; Dr. Wayland, Recording Secretary; Dr. D. Gregg, Treasurer; Drs. Dwinelle and Noble, Executive Committee; Drs. Ballard, Dwinelle and Blandy were appointed a committee to report at the next meeting on crystallized gold.

The following gentlemen were, on motion, appointed to read essays at the next annual meeting, viz. Drs. Gibbs, Ballard, Shepherd, Russell, Townsend, Harris and Austen. Prof. Blandy was chosen to deliver the opening address at the next meeting.

The Society adjourned to meet at 9 o'clock, A. M., February 28th, 1855, in the Chemical Hall of the Baltimore College of Dental Surgery.

SEN. ED.

---

*Curious Dental Pathological Specimens.*—Dr. W. H. Dwinelle recently presented to the senior editor, for the Museum of the Baltimore College of Dental Surgery, two bicuspids so perfectly united, that the root presents the appearance of that of but one tooth, while the two crowns, though united, are distinctly developed; one having the characteristics of an upper and the other that of a lower tooth. There is also in the collection, a specimen of osseous union of two supenumerary teeth, an upper cuspid with two fully developed roots and a superior molar, each of the roots of which are considerably enlarged by a deposit of osseous matter, but is not so intimately united to them but that it may be detached.

*Ossification of the Pulp of a Tooth.*—The junior editor received in a letter from Dr. Hubbard, a dentist of Kentucky, containing the pulp of a tooth in a state of ossification, an interesting example of which, furnished by Dr. F. H. Badger, of Tennessee, was described in a previous number of the Journal.

*Sugar in the Liver of Hybernating Animals.*—Professor Valentin, the eminent physiologist of Berne, has written a letter on this subject to Dr. Brinton, who has published an extract from it in the April No. of the ***British and Foreign Medico-Chirurgical Review.***

The liver of a male marmot, killed during his winter sleep, by confinement under an air-tight receiver, was boiled continuously with water. The filtered solution responded to all the tests of sugar. It caused an energetic reduction of oxyd of copper, acquired a deep yellow color by boiling with solution of potash, underwent an active fermentation when mixed with yeast, and rotated the plane of polarization towards the right. ***This animal had taken no food for more than six weeks.*** The quantity of sugar in the fresh liver was 2.87 per cent. of its total weight. Hepatic blood from the left lobe contained 0.85 per cent. of sugar. The bile and the grayish white gastric juice also were strongly impregnated with sugar. The diaphragm, the right suprarenal capsules and the muscular substance afforded slight reactions of sugar.

In a young hedgehog, in which hybernation was imperfect, and respiration frequent, no traces of sugar could be found. These two facts seem to point out a very close relation between respiration and the formation of sugar.

*Exodontosis, and a Lateral Incisor with Two Roots.*—A specimen of exodontosis, the size of a small hazlenut, and an upper lateral incisor, with two well developed roots, were recently presented to the senior editor, for the Museum of the Baltimore College of Dental Surgery, by Dr. Bretz, of Carlisle, Penn. On one side of the exostosis, about three lines from the apex of the root is a green spot which penetrates to a considerable depth in the substances of it, leading to the belief that the tooth had been filled with an amalgam containing copper. This seems to be the only way in which it can be accounted for.

---

*Improvements.*—To the members of the Dental Profession, and those engaged in the fabrication of articles used in the practice of dentistry.

The undersigned, chairman of a committee appointed by the Mississippi Valley Association, to meet and report improvements made during the current year, appertaining to the practice of dentistry, so far as the same may be made known, together with the names of the inventors or discoverers, is desirous of corresponding with those gentlemen who may have made any improvements they consider worthy of note, or discovered principles or things worthy the attention of the Association.

If the improvement is instrumental, or the discovery or principle is of such a nature as to be illustrated by specimens or drawings, these should accompany the description.

Specimens and illustrations will be preserved in the Society's cabinet, or returned if preferred.

The period for making the report is the 20th of February, 1855, communications should reach here before that time.

A. M. Leslie.

*Cinncinati, Dec.*

---

*Relations of the Liver and Spleen to Respiration.*—Liebig long ago advanced the notion that bile is an intermediate product of oxydation, its final results being water and carbonic acid. In order to establish this view, it would be desirable to show that the amount of carbonic acid, given off during respiration, is diminished when the liver is removed. To determine this matter, Moleschott has recently experimented upon frogs, deprived of their livers.

He used an ingenious apparatus, so contrived that air was admitted only in such quantities as were absolutely necessary to respiration, in which all the carbonic acid produced was absorbed by a potash apparatus.

He now weighed out 100 grammes of living frogs, unmutilated, and introduced them into the apparatus. On weighing his potash apparatus, he found that they had exhaled 430 milligrammes of carbonic acid in 24 hours. On substituting for sound frogs, 100 grammes of frogs without livers, he found that only 192 milligrammes were given off in the same length of time. Care was taken, in these comparative experiments, to select frogs as nearly as possible alike. He also found that the blood of these mutilated frogs contained no sugar, a fact which decidedly favors Bernard's views of the saccharifying power of the liver.

When the spleen was cut out, the relation of expired gas to that given off in the normal state was as 1 to 1 2-5, showing that it exerts much less influence upon the function of respiration than the liver.

---

*Our Present Number.*—The present number of the Journal has been delayed owing to failure on the part of several of our contributors to send in their articles at the specified time.

It will be observed that the Quarterly Summary is not embraced in this number. The reason is, that it has been crowded out by the original articles which have considerably exceeded their usual space.

With this number also commences not only a new volume, but a new arrangement by which hereafter the volume will begin with the year. This is more convenient, in many respects, and in order to accomplish it, we have omitted the October number. The only difference to our subscribers will be that the time of their subscriptions will be extended three months, so that those which would have expired with this number will be extended to April.

The next number will contain the Quarterly Summaries of Anatomy and Physiology, and of Chemistry, as well as of Dental Surgery. The Review Department will be carefully kept up, and we shall endeavor to present as large a variety as possible of meritorious original articles.

---

*Paralysis occasioned by the Extraction of a Tooth.*—A physician writes to the Editor of the Louisville Courier, that a few days ago, a young lady of Oldham county, Ky., in her ordinary health, perfectly well, the family say, rode two miles to a physician and had a tooth extracted. Almost immediately paralysis on one side of the body occurred, then stupor, and death followed in a few hours. She had not inhaled chloroform or anything of that kind.

